# Methionine restriction plus overload improves skeletal muscle and metabolic health in old mice on a high fat diet

**DOI:** 10.1038/s41598-021-81037-6

**Published:** 2021-01-13

**Authors:** Anandini Swaminathan, Andrej Fokin, Tomas Venckūnas, Hans Degens

**Affiliations:** 1 Institute of Sport Science and Innovations, Lithuanian Sports University, 44221 Kaunas, Lithuania; 2 Department of Life Sciences, Research Centre for Musculoskeletal Science and Sports Medicine, Manchester Metropolitan University, Manchester, UK

**Keywords:** Ageing, Physiology

## Abstract

Methionine restriction (MR) has been shown to reduce the age-induced inflammation. We examined the effect of MR (0.17% methionine, 10% kCal fat) and MR + high fat diet (HFD) (0.17% methionine, 45% kCal fat) on body mass, food intake, glucose tolerance, resting energy expenditure, hind limb muscle mass, denervation-induced atrophy and overload-induced hypertrophy in young and old mice. In old mice, MR and MR + HFD induced a decrease in body mass. Muscle mass per body mass was lower in old compared to young mice. MR restored some of the HFD-induced reduction in muscle oxidative capacity. The denervation-induced atrophy of the *m. gastrocnemius* was larger in animals on MR than on a control diet, irrespective of age. Old mice on MR had larger hypertrophy of *m. plantari*s. Irrespective of age, MR and MR + HFD had better glucose tolerance compared to the other groups. Young and old mice on MR + HFD had a higher resting VO_2_ per body mass than HFD group. Mice on MR and MR + HFD had a resting respiratory quotient closer to 0.70, irrespective of age, indicating an increased utilization of lipids. In conclusion, MR in combination with resistance training may improve skeletal muscle and metabolic health in old age even in the face of obesity.

## Introduction

With the worldwide increasing ageing population, it is essential to study ways to improve their quality of life. Two major health concerns faced by the older population are sarcopenia and obesity. Sarcopenia is defined as the loss of skeletal muscle mass and function during ageing^1^ and obesity is defined as the accumulation of fat^2^. The age-related reduction in muscle mass and function can further be exacerbated by obesity, where adipose tissue can release inflammatory cytokines that contribute to muscle wasting^3^. Indeed, the physiological consequences of obesity act as risk factors for the development of sarcopenia^4^.

Reduced energy expenditure (EE) as a result of lower physical activity and basal metabolic rate are associated with ageing and are risk factors for the development of sarcopenia^5,6^. In addition to reduced EE due to lowered physical activity levels, also the age related loss of muscle mass decreases metabolic rate and further contributes to a decrease in physical activity. Both factors contribute to the gain in fat mass that in turn induces loss of muscle mass via release of inflammatory cytokines^7^. Furthermore, obesity and elevated levels of circulating free fatty acids^8^ may cause resistance to anabolic stimuli like insulin^9^, growth factors, hormones, amino acids and exercise—a phenomenon known as anabolic resistance^10^. The accumulation of intramuscular fat as seen in obesity, can also lead to anabolic resistance by reducing phosphorylation of targets in the mTOR, AMPK pathways^11^ and thereby increase the risk of sarcopenia^12–14^.

While all the interactions between obesity and sarcopenia are not fully understood, it is clear that dietary intervention and exercise are essential to counteract the loss of muscle mass of obese people with sarcopenia. Calorie restriction without malnutrition^15^ is a popular intervention against obesity, and resistance training helps build muscle mass even in very old people^16^ and improve body composition^17^. While calorie restriction yields desirable results in terms of combatting obesity^18^, it is challenging to limit food intake over a long period of time. Methionine restriction (MR) has emerged as a promising mimetic to caloric restriction because it is associated with loss of body mass, improved metabolic profile and longevity without having to reduce food intake^15,19,20^. It has also been reported to improve glucose tolerance, increase energy expenditure (EE), limit fat deposition and to increase de novo synthesis of hepatic triglycerides^21^. While it has been established that MR increases lifespan and lowers inflammation during ageing^22^, not much is known about the effect of MR on skeletal muscle during ageing and how it affects the atrophic and hypertrophic response. Therefore, the aim of the present study was to assess the effects of MR on skeletal muscle mass and plasticity in young and old mice subjected to an obesogenic high-fat diet. We hypothesized that MR will improve body composition in both young and old mice by reducing body mass, increasing muscle mass, improve the hypertrophic response to an overload stimulus and reduce atrophy in response to denervation, and improve glucose tolerance even in the presence of high fat intake. If so, this will make MR a suitable intervention for the elderly post-hospitalization and bed rest.

## Results

### Body mass

**Figure 1:**
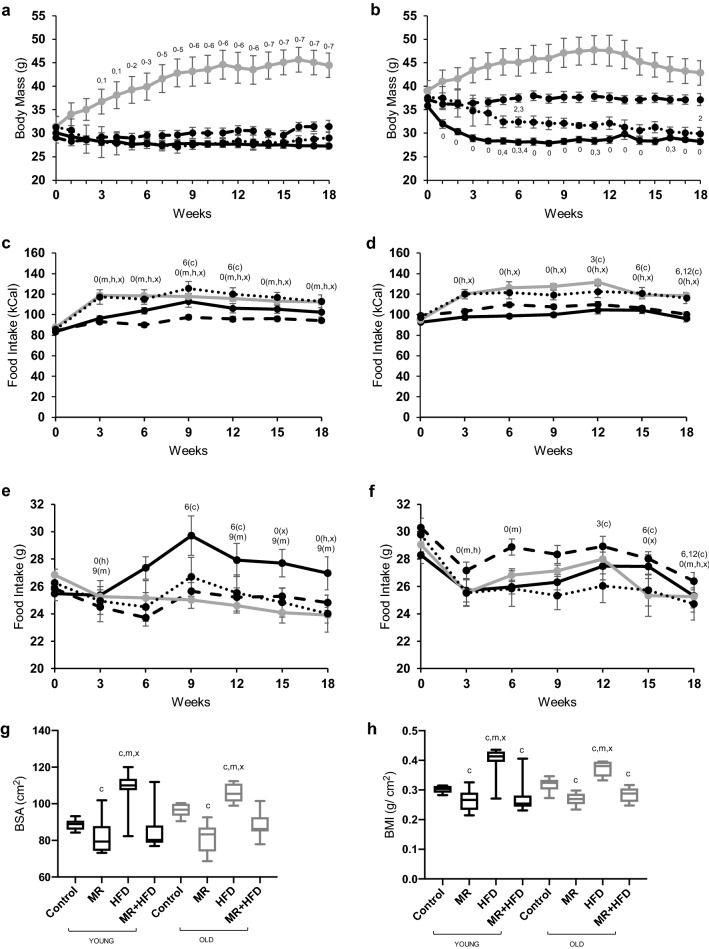
Body mass (g) (**a**,**b**), food intake in kCal (**c**,**d**) and grams (**e**,**f**), body surface area (**g**), body mass index (BMI) (**h**) in young (**a**,**c**,**e**) and old (**b**,**d**,**f**) mice for 18 weeks on a control (dash line), methionine restricted (MR) (solid black line), high fat diet (HFD) (grey line) or MR + HFD (dotted line) diet. 0, 1, 2, 3, 4, 5, 6, 7, 9: significantly different from weeks 0, 1, 2, 3, 4, 5, 6, 7, 9 respectively; c, m, x: significantly different from control, MR, MR + HFD group, respectively; for food intake (**c**–**f**) c, m, h, x: control, MR, HFD, MR + HFD, respectively, represent the diet groups in which these differences are observed all at p < 0.05. Data is presented as mean ± SEM (**a**–**f**) and mean, min, max (**g**,**h**).

At baseline the old mice had a higher body mass than the young mice (p < 0.001). There were significant main effects of time, age and diet on body mass over the 18-week period of the respective diets (all p < 0.001). As there were also time × age and time × diet interactions (p < 0.001) on body mass, post-hoc tests were done in each age and diet group separately. Post-hoc tests revealed no significant differences in body mass over time in young (Fig. 1a) and old (Fig. 1b) animals on a control diet. MR induced a decrease in body mass in old (p ≤ 0.027; Fig. 1b), but not in young mice (Fig. 1a). HFD induced an increase in body mass in young mice (p ≤ 0.036, Fig. 1a) and there was a trend towards significant increase in body mass in the old group (p = 0.056; Fig. 1b). In young mice (Fig. 1a) on MR + HFD, there was no significant change in body mass, but in the old mice on MR + HFD body mass decreased (p ≤ 0.037; Fig. 1b).

### Calorie intake and food intake

Figure 1c,d show the caloric intake over the 18-week period of young and old mice, respectively. There were main effects of age (p = 0.012), diet (p < 0.001) and time (p < 0.001) on caloric intake and time × diet (p < 0.001) and time × age (p = 0.011) interactions. To investigate this further, in each age and diet group, post-hoc tests over time were done. Before switching to the different diets, the caloric intake was higher in old than young mice (p < 0.001). Young mice on the control diet consumed more calories at weeks 9 and 12 than at week 6 (p ≤ 0.004; Fig. 1c), while the old mice on the control diet consumed the most calories at weeks 3 and 6 (p ≤ 0.048; Fig. 1d). While young mice on a MR diet had a higher calorie intake at week 18 than week 0 (p ≤ 0.042; Fig. 1c), there was no significant change in the calorie intake of old mice on MR (Fig. 1d). The calorie intake in the HFD and the MR + HFD at week 18 was greater than week 0, irrespective of age (p ≤ 0.001; Fig. 1c,d).

Figures 1e,f show the food intake in grams over the 18-week period of young and old mice, respectively. The food intake at the start of the experiment (week 0) was higher in old than young mice (p = 0.006). There were significant main effects of age (p < 0.001), diet (p = 0.005) and time (p < 0.001), and time × diet (p = 0.044) and time × age (p < 0.001) interactions for food intake. Therefore in each age group and diet group, post hoc tests over time were done. The food intake on the control diet was transiently higher at week 9 and 12 than at week 6 in the young mice (p ≤ 0.004; Fig. 1e), while in the old animals the food intake was transiently highest at 12 weeks (p ≤ 0.049; Fig. 1f). Young animals on MR had a transiently elevated food intake at week 9 (Fig. 1e), while in old animals the food intake was reduced (Fig. 1f). Both young (Fig. 1e) and old (Fig. 1f) mice on a HFD had a lower food intake at week 18 than at week 0 (p < 0.01). A similar pattern was found for animals on MR + HFD (p ≤ 0.011).

Overall, MR induced a transient increase in food intake in young mice, but their caloric intake was similar. Mice on HFD and MR + HFD, on the other hand, consumed less food but more calories by week 18.

### Body surface area (BSA), body mass index (BMI)

Figure 1g,h show the impacts of the different 18-week diets on the BSA and BMI, respectively. There was no significant difference in BSA and BMI between young and old mice (p = 0.298 and p = 0.955, respectively). Mice on a HFD had, however, a higher BSA and BMI than those on control, MR, or HFD + MR diet (p < 0.001). Mice on MR had a lower BSA than the control group (p < 0.001; Fig. 1g) and the BMI was lower than the controls in both MR and MR + HFD mice (p ≤ 0.041; Fig. 1h), irrespective of age.

In summary, it appears that MR in the presence or absence of HFD leads to decreased body mass in old mice and lower BMI in both age groups.

### Metabolic profile

Table 1 shows energy expenditure (EE), EE per body mass, resting VO_2_ and resting VO_2_ per body mass in young and old mice. Energy expenditure was greater in old compared to young mice (p = 0.025), and animals on MR had lower EE compared to those on HFD (p = 0.012) irrespective of age. There were no significant effects of age (p = 0.943) or diet (p = 0.105) on EE per body mass. There was no main age effect on resting VO_2_, but post hoc tests showed lower resting VO_2_ in mice on MR than HFD (p = 0.002), irrespective of age. Resting VO_2_ per body mass was not significantly influenced by age, but was higher in the MR + HFD than HFD group (p < 0.001), irrespective of age. Figure 5c shows that the resting RQ did not differ significantly between young and old mice (p = 0.725). The RQ was, however, lower in the HFD and MR + HFD groups compared to control group (p ≤ 0.001) irrespective of age.

**Table 1:** Resting oxygen uptake (VO_2_), resting VO_2_ per body mass, energy expenditure (EE) and EE per body mass in young and old mice fed—control, methionine restricted (MR), high fat diet (HFD), or MR + HFD.

	Young				Old			
	Control	MR	HFD	MR + HFD	Control	MR	HFD	MR + HFD
Resting VO_2_ (mL min^−1^)	1.60 ± 0.08	1.50 ± 0.08^h^	1.90 ± 0.08	1.60 ± 0.15	1.90 ± 0.08	1.50 ± 0.15^h^	2.00 ± 0.08	1.90 ± 0.15
Resting VO_2_ (mL min^−1^ g^−1^)	0.053 ± 0.003	0.055 ± 0.002	0.046 ± 0.003	0.062 ± 0.004^h^	0.050 ± 0.002	0.052 ± 0.004	0.045 ± 0.003	0.059 ± 0.004^h^
EE (kCal min^−1^)	11.4 ± 0.6	10.6 ± 0.6^h^	13.0 ± 0.5	9.5 ± 1.7	13.5 ± 0.6^y^	10.2 ± 0.9^h,y^	13.6 ± 0.6^y^	13.0 ± 0.9^y^
EE (kCal min^−1^ g^−1^)	0.37 ± 0.02	0.38 ± 0.01	0.31 ± 0.02	0.36 ± 0.06	0.35 ± 0.01	0.36 ± 0.03	0.31 ± 0.02	0.40 ± 0.03

^h^Significantly different from HFD at p ≤ 0.012.

^y^Significantly different from young mice at p = 0.025.

### Glucose tolerance

**Figure 2:**
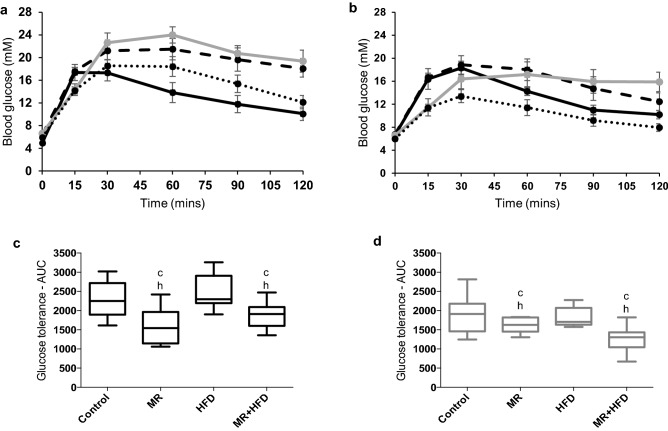
Glucose tolerance. Blood glucose concentrations (mM) measured at 0, 15, 30, 60, 90, 120 min after an intraperitoneal injection of 2 g glucose per kg body mass glucose in (**a**) young and (**b**) old mice fed control, methionine restricted (MR), high fat diet (HFD), or MR + HFD diet. The area under the curve calculated from glucose concentrations in (**c**) young and (**d**) old mice and c, h: significantly different from control and HFD groups respectively at p ≤ 0.003. Data is presented as (**a**,**b**) mean, min, max and (**c**,**d**) mean ± SEM.

Figure 2a,b show the time course of changes in blood glucose concentrations after a bolus injection of glucose. It can be seen in Fig. 2c,d that, irrespective of age, animals on MR and MR + HFD had a better glucose tolerance compared to the control and HFD groups (p ≤ 0.003).

### Muscle mass

**Figure 3:**
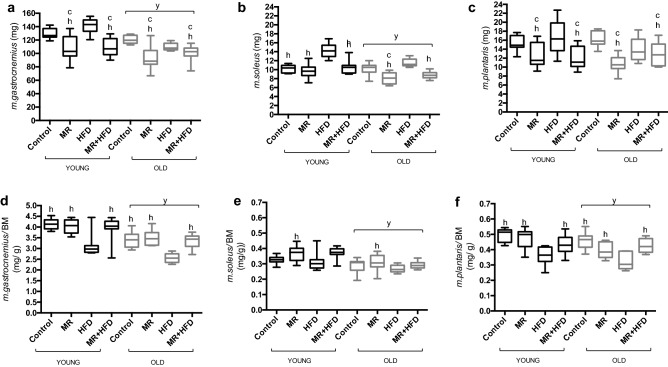
(**a**) *m. gastrocnemius,* (**b**) *m. soleus,* (**c**) *m. plantaris* mass (mg), (**d**) *m. gastrocnemius* mass per body mass (BM), (**e**) *m. soleus* mass per body mass and (**f**) *m. plantaris* mass per body mass (mg/g) from the control leg of young (black boxes) and old (grey boxes) mice fed—control, methionine restricted (MR), high fat diet (HFD) and MR + HFD. y: significantly different from young at p ≤ 0.009. c, h: significantly different from control and HFD respectively at p ≤ 0.026. Data is presented as mean, min, max.

Figure 3 shows the mass of control *m. gastrocnemius* (Fig. 3a), *m. soleus* (Fig. 3b) and *m. plantaris* (Fig. 3c) in young and old mice fed control, MR, HFD and MR + HFD diets. The mass of the *m. gastrocnemius* was higher in young than old animals (p < 0.001). The MR and MR + HFD groups had a lower *m. gastrocnemius* mass compared to the control and HFD groups (p < 0.001), irrespective of age.

Young animals had a larger *m. soleus* mass than old (p < 0.001; Fig. 3b). There was, however, an age × diet interaction (p = 0.030) for the *m. soleus* mass. In the young animals the mass of the soleus was higher in mice on a HFD than that in control, MR and MR + HFD groups (p < 0.001). In old animals the soleus muscle mass was also higher in animals on a HFD than on MR and MR + HFD (p < 0.001), but there was no significant difference in soleus mass between mice on a HFD and control diet. In the old animals only, the MR animals had a lower soleus muscle mass than control and HFD groups (p ≤ 0.003).

There was no significant difference in *m. plantaris* mass between young and old animals (p = 0.255; Fig. 3c). The MR and MR + HFD groups both had lower *m. plantaris* mass than the control and HFD groups (p ≤ 0.005), irrespective of age.

Muscle mass per body mass indicated that old mice had significantly lower *m. gastrocnemius* (Fig. 3d), *m. soleus* (Fig. 3e) and *m. plantaris* (Fig. 3f) mass compared to the young (p ≤ 0.009). The *m. gastrocnemius* and *m. plantaris* mass per body mass were higher in animals fed control, MR and MR + HFD compared to the HFD group (p < 0.001) irrespective of age. Only MR fed mice had greater *m. soleus* mass per body mass compared to the HFD group (p = 0.026) irrespective of age.

**Figure 4:**
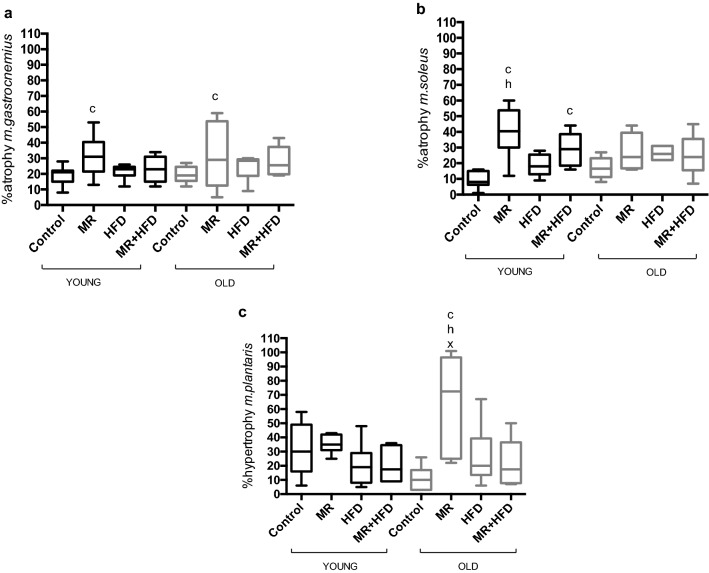
(**a**) % atrophy *m. gastrocnemius,* (**b**) % atrophy *m. soleus* and (**c**) % hypertrophy *m. plantaris* of young (black boxes) and old (grey boxes) mice fed—control, methionine restricted (MR), high fat diet (HFD) and MR + HFD. c, h, x: significantly different from control, HFD and MR + HFD respectively at p ≤ 0.027. Data is presented as mean, min, max.

Figure 4a shows that age did not have a significant effect on % atrophy of the *m. gastrocnemius* (p = 0.430). The % atrophy was larger in animals on MR than on a control diet (p = 0.009), irrespective of age.

Age did not have a significant effect on % atrophy (p = 0.695) of the *m. soleus* but there was a significant effect of diet (p < 0.001; Fig. 4b) and an age × diet interaction (p = 0.023). Further analyses revealed that in young mice on MR diet the % atrophy was larger than in control and HFD mice (p ≤ 0.002) and in MR + HFD fed mice % atrophy was larger than in mice on a control diet (p ≤ 0.003). In old mice, however, diet did not have significant effect on the % atrophy (p = 0.214).

Figure 4c shows that there was no significant age effect (p = 0.395), but there was a diet effect and an age × diet interaction (p ≤ 0.008) for the % hypertrophy of the *m. plantaris*. While there was no significant effect of diet on % hypertrophy in young mice (p = 0.175), in the old, animals on MR had larger % hypertrophy as compared to the control, HFD and MR + HFD groups (p ≤ 0.027).

Briefly, both old and young mice on MR and MR + HFD appear to have lower muscle mass. MR resulted in a more pronounced denervation-induced atrophy in young mice and a more pronounced hypertrophic response in old mice.

### SDH

**Figure 5:**
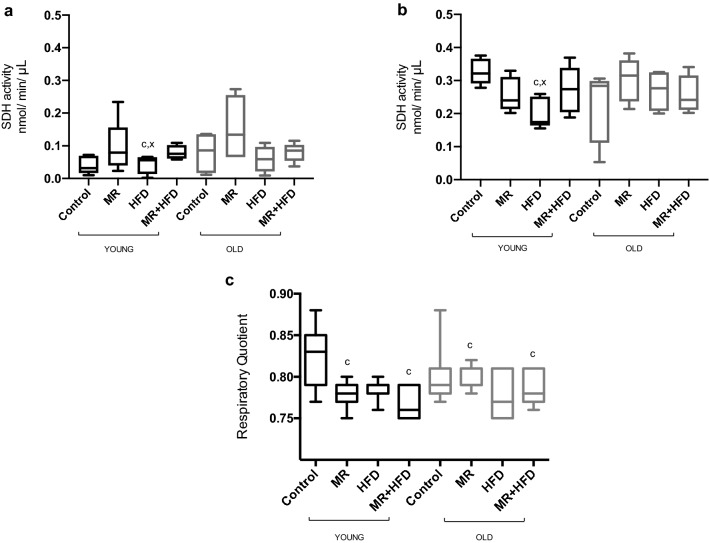
Succinate dehydrogenase (SDH) activity in (**a**) *m. gastrocnemius* and (**b**) *m. soleus*; (**c**) respiratory quotient (RQ) in young (black boxes) and old (grey boxes) mice fed—control, methionine restricted (MR), high fat diet (HFD), MR + HFD. For SDH activity (**a**,**b**): c: significantly different from control and MR + HFD in young mice at p ≤ 0.020. For RQ (**c**): c: significantly different from control in (**b**) young and (**c**) both young and old mice at p ≤ 0.011. Data is presented as mean, min, max.

The SDH activity was lower in the *m. gastrocnemius* (Fig. 5a) than in the *m. soleus* (Fig. 5b) (p < 0.001). While there was no main effect of age or diet, there was an age × diet interaction (p = 0.021) so post hoc tests were done separately for young and old groups. This revealed that the SDH activity in muscles from young mice fed a HFD was lower than that in mice on a control diet or MR + HFD (p ≤ 0.020; Fig. 5a,b), while there was no significant effect of diet on muscle SDH activity in old mice.

## Discussion

Here, we investigated the impact of MR in young and old mice on a normal or high fat diet on body mass, food intake, glucose tolerance, resting energy expenditure, SDH activity, hind limb muscle mass and denervation-induced atrophy and overload-induced hypertrophy. The main observation is that MR abolished the HFD-induced increase in body mass, BMI and decline in muscle mass to body mass ratio. MR also resulted in an enhanced glucose tolerance even when combined with a HFD, indicative of enhanced insulin sensitivity. In addition, MR enhanced the hypertrophic response. These benefits of MR were observed in both young and old mice. However, the effects of MR were not unequivocally beneficial, as MR accentuated denervation-induced atrophy in both the young-adult and old mice. These data suggest that MR may be beneficial to combat the adverse effects of a HFD on sarcopenia, but it may be detrimental during periods of disuse such as during spaceflight, hospitalisation and prolonged bed rest, especially in older adults.

It has been reported that the food intake declines during ageing^23–25^. In contrast to humans, however, the food intake before changing the diets was higher in old than young mice. Nevertheless, similar to the observations in humans, the body mass was higher in old than young mice. However, in contrast to the higher BMI in old people^26^, this higher body mass did not translate into a higher BSA or BMI in the old compared to the young mice. Despite the similar BMI and BSA in old and young mice, the *m. gastrocnemius*, *m. soleus* and *m. plantaris* mass per body mass was lower in old animals, indicating that also in mice there is an age-related reduction in the proportion of lean mass. In line with this, it has been seen that visceral and gonadal fat mass was higher in old than young mice^27,28^.

Both quantity of food intake and diet composition are key determinants of body composition. It is interesting to note that although a HFD led to a lower amount of food intake, the caloric intake was elevated in both young and old mice. Similar to previous observations the higher caloric intake was accompanied by an increase in body mass^29^. It was somewhat surprising, however, that the increase in body mass was greater in young than old mice, as in a previous study old mice had a larger gain in body mass^29^. The discrepancy may be attributable to the duration of HFD, as it has been observed that old mice become more easily obese than young in response to a short-term HFD, whereas chronic HFD feeding causes greater body mass increase in young than old mice^30^.

Mice on a MR diet, on the other hand, consumed the same amount of food as the animals on a control diet, as has also been reported by others^31,32^, yet lost body mass and had a reduced BMI. Yet, the muscle mass to body mass ratio was similar to mice on a control diet, suggesting that the MR-induced loss of body mass was due to a proportional loss of muscle mass and fat. MR did, however, prevent the HFD-induced reduction in muscle mass to body mass ratio in both young and old mice. This is in accordance with a previous observation that MR protected against the development of obesity in young mice on a HFD^33^. Our data indicate that this protection against HFD-induced obesity by MR also occurs in old age.

The loss of body mass despite increased food intake has been linked to metabolic inefficiency created by MR which leads to increased EE^34^ through uncoupling protein 1 (UCP1)-related non-shivering thermogenesis in adipose tissue^31^. Although this is an attractive hypothesis, it does not explain the MR-induced loss of body mass in our study as we did not observe an increase in EE.

It is possible that the loss of body mass may be attributable to MR-induced lipolysis^35^, also in skeletal muscle, which would then explain the reduction in body mass even when a HFD is combined with MR.

The RQ provides a real-time index of substrate utilization during the metabolic cycle and is based on the molar ratios of O_2_ consumed and CO_2_ produced during the oxidation of glucose (1.00), lipid (0.70) and protein (0.80)^36,37^. Here, we observed that both young and old mice in the MR and MR + HFD groups had RQ closer to 0.70 compared to control-fed mice with RQ > 0.80. This shift towards an increased utilization of lipids by MR fed animals was also observed by^31^, and corresponds with the suggested MR-induced lipolysis^35^.

Somewhat unexpected is the observation that HFD did not impair glucose tolerance in either young or old mice, given that a HFD is a risk factor for the development of insulin resistance^38,39^. It is possible that the duration of the HFD plays a role, as it has been observed that the glucose intolerance was more pronounced after 12 than 4 weeks of HFD^40^ and at least in young-adult mice the accumulation of intramyocellular fat did occur after 16 weeks, but not after 8 weeks on a HFD^29^. Other studies, however, have seen impaired insulin sensitivity as soon as after 8 weeks of a HFD^41^ that may result in impaired glucose tolerance. It should be noted, however, that the HFD duration in our study was longer (16 weeks) than the longer durations of HFD (12 weeks) in the other studies^29,40^ and we have no explanation for the discrepancy between studies. It remains to be seen whether the maintained glucose tolerance in our study was realised by enhanced insulin secretion after a HFD.

Our data showed that young and old mice on MR were more glucose tolerant than control mice on a control diet, irrespective of the fat intake. This indicates that MR improves the metabolic profile even when fat intake is high. Similar observations were made in young mice on MR^42^ and MR + HFD^33^ compared to control-fed mice after 8 weeks of dietary intervention. It has been reported that even just 48 h MR improved glucose tolerance^42^. This may be attributable to enhanced glycogen synthesis, glycolysis and aerobic oxidation in the skeletal muscle of mice on MR and MR + HFD^43^. Indeed, we observed that MR restriction prevented or attenuated the HFD-induced reduction in SDH activity in the *m. soleus* and *m. gastrocnemius* of both young and old mice. These data indicate that MR may be an effective intervention to improve glucose tolerance in obesity and old age.

The effects of MR on skeletal muscle mass have not been fully studied. While the muscle mass to body mass ratio was lower in old than young mice, there was no significant difference in absolute muscle mass. Interestingly, denervation resulted in a similar final mass in young and old mice. Such an observation was previously been reported in young and old rats^44,45^, and it was suggested that denervation may cause the muscle to atrophy to a sort of ‘default’ muscle mass.

Somewhat unexpected was the absence of an attenuated hypertrophic response in the old mice that had been reported in mice previously^46^. It is unlikely that this discrepancy is attributable to the higher age of our animals than those in the study by^46^ as it has been shown that particularly at high age the hypertrophic response is blunted^47^. It is possible that the apparent absence of a significantly attenuated hypertrophy in the older mice is related to the small sample size and the modulation of the hypertrophic response by the dietary interventions that potentially obscured any age effects in our study. In support of this, the data in the mice on a control diet are in the direction of an attenuated hypertrophic response in the old animals.

Here we observed that MR induces a loss of muscle mass in young and old animals that is proportional to the decrease in body mass. While the denervation-induced atrophy was aggravated with MR, the hypertrophic response was enhanced in the old animals only, irrespective of concurrent HFD. Since hypertrophy and angiogenesis follow a similar time course^48^ and the blunted hypertrophy in old mice was associated with impaired angiogenesis^46^, we speculate that the enhanced hypertrophic response in the old MR-fed mice may be attributable to enhanced angiogenesis. Indeed, it has been observed that amino acid restriction promotes angiogenesis via upregulation of vascular endothelial growth factor (VEGF) that via H_2_S activates AMPK^49^, or upregulates SIRT1^50^ that both enhance angiogenesis also in old age. Combining these findings, we suggest that MR promotes angiogenesis via VEGF-induced upregulation of AMPK and SIRT1, resulting in an enhanced hypertrophic response in old age.

In conclusion, MR promotes glucose tolerance and results in a decrease in body mass and BMI, even in the presence of a HFD. It also enhanced the hypertrophic response. Unexpectedly, however, MR aggravated the denervation-induced atrophy. These effects of MR applied to both young and old mice. Though speculative at the moment, these observations suggest that a combination of methionine restriction and resistance training may benefit the sarcopenic elderly population, but methionine restriction should not be applied during periods of bed rest such as during hospitalisation.

## Methods

All experiments were approved by the ethics committee of the Lithuanian Republic Alimentary and Veterinary Public Office (No. G2-90 in 2018) and carried out in accordance with the guidelines and regulations stated. Male C57BL/6J mice were housed individually at 20–22 °C in a 12 h light/dark cycle at the animal research facility at the Lithuanian Sports University. Animals had free access to water and standard chow until the age of 6 (young-adult mice, n = 38) or 22 months (old mice, n = 32). Then they were sub-divided into the following groups: control, methionine restricted (MR), high fat diet (HFD) and MR + HFD. Diets were purchased from Research Diets Inc. (New Brunswick, NJ, USA) and the diet compositions are shown in Table 2. Animals were allowed ad libitum access to food and water and the body mass and food intake were monitored weekly from one week prior to starting the experimental diet until terminal experiments.

**Table 2:** Diet composition of chow and experimental diets fed to mice for the duration of the study.

Diet	%Carbohydrate	%Protein	%Fat	%Methionine
Chow	66	21	6	0.65
Control	72	18	10	0.49
MR	72	17	10	0.17
HFD	36	18	46	0.61
MR + HFD	37	17	46	0.17

Nutrients are expressed as percentages of total calories (kCal).

*MR* methionine restricted diet, *HFD* high fat diet.

### Denervation

At the age of 8.5 (young) or 25.5 (old) months compensatory hypertrophy of the right plantaris muscles was induced in all mice by cutting the branches of the *n. Ischiadicus* supplying the *m. gastrocnemius* and *m. soleus* as close to their point of entry to the belly of the muscle and a portion of each branch was removed to prevent reinnervation. Surgery was performed under anaesthesia (isoflurane − 4% and O_2_ at 2 L/min until the animal did not respond to foot-pad-pinch and then maintained with 1.5% isoflurane and 1 L/min O_2_) under aseptic conditions. Hypertrophy of the *m. plantaris* was expected 6 weeks post the denervation surgery^46^.

### Glucose tolerance

A week prior to the terminal experiment, glucose tolerance was determined. Thereto, the animals were fasted overnight (16 h) and then subjected to an intraperitoneal injection of glucose (2 g glucose/kg body mass). A glucometer (Glucocard X-mini plus, Japan) was used to measure blood glucose from blood samples taken from an incision made in the tail vein before (0 min) and at 15, 30, 60, 90 and 120 min after the injection. Prism 7.0 software was used to calculate the area under the glucose—time curve (AUC).

### Metabolic measurements

Metabolic measurements were performed during the light cycle (0900–1800) as described in another study by our group^51^. Before being transferred to the metabolic cage, the body mass of the mouse was determined on a scale (440–45 N, Kern, Germany). The energy expenditure (EE), VO_2_ and the respiratory quotient (RQ) were assessed over a 3-h period in a Panlab metabolism system (Physiocage, Panlab Harvard Apparatus, Spain) equipped with a standard-size metabolic cage. During the measurement, mice had free access to water but not food. The metabolic cage was connected to a gas analyser (LE405, Panlab Harvard Apparatus, Spain) with a switching device for control of airflow (LE400, Panlab Harvard Apparatus, Spain). Before the experiment, the gas analyser was calibrated at a high point (50% O_2_, 1.5% CO_2_) and low point (20% O_2_, 0% CO_2_). The airflow was set at 250 mL/min and switched every 3 min to assess O_2_ and CO_2_ concentrations in the metabolic cage and the external environment. The data of the first hour were discarded, as the first hour was a period of acclimation to the metabolic cage and the data of the last 2 h averaged. After the measurement, the animals were transferred back to their cages.

### Terminal experiment

At 10 (young) or 27 (old) months, animals were euthanised with an overdose of CO_2_. Hind limb muscles—*m. gastrocnemius*, *m. plantaris* and *m. soleus*—were carefully excised, weighed, frozen in isopentane pre-cooled with liquid nitrogen, and stored at − 80 °C until further analysis.

Body mass (BM) and nasoanal (NAL) length were used to calculate body mass index (BMI)^52^ and body surface area (BSA)^53^ with the equations below.

1 $$Body \,mass \,index \left(\text{g }\cdot{\text{cm}}^{-2}\right)=\frac{BM\,\left(\text{g}\right)}{NAL\,{(\text{cm})}^{2}}$$

2 $$Body\, Surface \,area \left({\text{cm}}^{2}\right)=71.84 x BM\,\left({\text{g}}^{0.425}\right) \times NAL\,({\text{cm}}^{0.725})$$

### Succinate dehydrogenase (SDH) activity

We used the method to assess SDH activity described previously^54,55^. Briefly, 10 mg of skeletal muscle (*m. gastrocnemius* or *m. soleus*) tissue was homogenised in 100 μL ice cold lysis buffer (50 mM Tris–HCl, 1 mM ethylene diamine tetra acetic acid, 1 mM ethylene bis (oxyethylene nitrilo) tetra acetic acid, 50 mM sodium fluoride, 1 mM sodium orthovanadate, 10 mM β-glycerophosphate, 1% Triton X-100, pH adjusted to 7.0). The homogenate was kept overnight at − 80 °C and centrifuged the next day at 13,000×*g* for 10 min. 20 μL of the supernatant was added to 96-well plates which contained 180 μL reaction reagent (50 mM NaPi buffer (pH 7.4), 1 mM KCN, 0.06 mM 2,6-DCPIP, 0.2% (wt/vol) bovine serum albumin) and 20 μL 100 mM sodium succinate solution. The change in absorbance at 600 nm per minute was measured using a spectrophotometric plate reader (Spark 10 M, Tecan Group Ltd, Zürich, Switzerland).

### Statistics

Data are presented as mean ± SEM or mean, min, max. To determine the changes in body mass and food intake over time, a repeated-measures ANOVA with time as within factor and age and diet as between factors. A repeated-measures ANOVA was also performed to determine changes in SDH activity with muscle as within factor and age and diet as between factors. One-way ANOVA was used to test for differences in other body composition measures, muscle mass, effects of denervation and measures of metabolic profile with age, diet and where appropriate denervation, as factors. Effects were considered significant at p < 0.005. All calculations were performed using IBM SPSS Version 23.

## Abbreviations

MR: Methionine restriction
HFD: High fat diet
CR: Calorie restriction
EE: Energy expenditure
RQ: Respiratory quotient
SDH: Succinate dehydrogenase

## References

[1] Rosenberg IH (1989). Summary comments: Epidemiological and methodological problems in determining nutritional status of older persons. Am. J. Clin. Nutr..

[2] Garrow JS (1988). Obesity and related diseases. Appetite..

[3] Kalinkovich A, Livshits G (2017). Sarcopenic obesity or obese sarcopenia: A cross talk between age-associated adipose tissue and skeletal muscle inflammation as a main mechanism of the pathogenesis. Ageing Res. Rev..

[4] Rolland Y (2008). Sarcopenia: Its assessment, etiology, pathogenesis, consequences and future perspectives. J. Nutr. Health Aging.

[5] Elia M, Ritz P, Stubbs RJ (2000). Total energy expenditure in the elderly. Eur. J. Clin. Nutr..

[6] Cooper JA (2013). Longitudinal change in energy expenditure and effects on energy requirements of the elderly. Nutr. J..

[7] Wang J, Leung KS, Chow SKH, Cheung WH (2017). Inflammation and age-associated skeletal muscle deterioration (sarcopaenia). J. Orthop. Transl..

[8] Lang CH (2006). Elevated plasma free fatty acids decrease basal protein synthesis, but not the anabolic effect of leucine, in skeletal muscle. Am. J. Physiol. Endocrinol. Metab..

[9] Guillet C (2009). Changes in basal and insulin and amino acid response of whole body and skeletal muscle proteins in obese men. J. Clin. Endocrinol. Metab..

[10] Nilsson MI (2013). Abnormal protein turnover and anabolic resistance to exercise in sarcopenic obesity. FASEB J..

[11] Mounier R (2011). Antagonistic control of muscle cell size by AMPK and mTORC1. Cell Cycle.

[12] Hilton TN, Tuttle LJ, Bohnert KL, Mueller MJ, Sinacore DR (2008). Excessive adipose tissue infiltration in skeletal muscle in individuals with obesity, diabetes mellitus, and peripheral neuropathy: Association with performance and function. Phys. Ther..

[13] Tardif N (2014). Muscle ectopic fat deposition contributes to anabolic resistance in obese sarcopenic old rats through eIF2α activation. Aging Cell.

[14] Cleasby ME, Jamieson PM, Atherton PJ (2016). Insulin resistance and sarcopenia: Mechanistic links between common co-morbidities. J. Endocrinol..

[15] Orgeron ML (2014). The impact of dietary methionine restriction on biomarkers of metabolic health. Prog. Mol. Biol. Transl. Sci..

[16] Harridge SDR, Kryger A, Stensgaard A (1999). Knee extensor strength, activation, and size in very elderly people following strength training. Muscle Nerve.

[17] Faulks SC, Turner N, Else PL, Hulbert AJ (2006). Calorie restriction in mice: Effects on body composition, daily activity, metabolic rate, mitochondrial reactive oxygen species production, and membrane fatty acid composition. J. Gerontol. A Biol. Sci. Med. Sci..

[18] Schübel R (2018). Effects of intermittent and continuous calorie restriction on body weight and metabolism over 50 wk: A randomized controlled trial. Am. J. Clin. Nutr..

[19] Orentreich N, Matias JR, DeFelice A, Zimmerman JA (1993). Low methionine ingestion by rats extends life span. J. Nutr..

[20] Perrone CE, Malloy VL, Orentreich DS, Orentreich N (2013). Metabolic adaptations to methionine restriction that benefit health and lifespan in rodents. Exp. Gerontol..

[21] Forney LA, Wanders D, Stone KP, Pierse A, Gettys TW (2017). Concentration-dependent linkage of dietary methionine restriction to the components of its metabolic phenotype. Obesity.

[22] Wanders D (2018). The components of age-dependent effects of dietary methionine restriction on energy balance in rats. Obesity..

[23] Morley JE, Silver AJ (1988). Anorexia in the elderly. Neurobiol. Aging.

[24] Silver AJ, Guillen CP, Kahl MJ, Morley JE (1993). Effect of aging on body fat. J. Am. Geriatr. Soc..

[25] Aloia JF, Vaswani A, Ma R, Flaster E (1996). Aging in women—The four-compartment model of body composition. Metabolism.

[26] Mcphee JS (2018). Biological sciences cite as. J. Gerontol. A Biol. Sci. Med. Sci..

[27] Tallis J, Hill C, James RS, Cox VM, Seebacher F (2017). The effect of obesity on the contractile performance of isolated mouse soleus, EDL, and diaphragm muscles. J. Appl. Physiol..

[28] Hill C, James RS, Cox VM, Tallis J (2019). Does dietary-induced obesity in old age impair the contractile performance of isolated mouse soleus, extensor digitorum longus and diaphragm skeletal muscles?. Nutrients.

[29] Messa GAM (2020). The impact of a high-fat diet in mice is dependent on duration and age, and differs between muscles. J. Exp. Biol..

[30] Okada T, Mita Y, Sakoda H, Nakazato M (2019). Impaired adaptation of energy intake induces severe obesity in aged mice on a high-fat diet. Physiol. Rep..

[31] Hasek BE (2010). Dietary methionine restriction enhances metabolic flexibility and increases uncoupled respiration in both fed and fasted states. Am. J. Physiol. Regul. Integr. Comp. Physiol..

[32] Plaisance EP (2011). Dietary methionine restriction increases fat oxidation in obese adults with metabolic syndrome. J. Clin. Endocrinol. Metab..

[33] Ables GP, Perrone CE, Orentreich D, Orentreich N (2012). Methionine-restricted C57BL/6J mice are resistant to diet-induced obesity and insulin resistance but have low bone density. PLoS ONE.

[34] Bárcena C (2018). Methionine restriction extends lifespan in progeroid mice and alters lipid and bile acid metabolism. Cell Rep..

[35] Cooke D (2020). Weight loss and concomitant adipose autophagy in methionine-restricted obese mice is not dependent on adiponectin or FGF21. Obesity.

[36] Ferrannini E (1988). The theoretical bases of indirect calorimetry: A review. Metabolism.

[37] Simonson DC, DeFronzo RA (1990). Indirect calorimetry: Methodological and interpretative problems. Am. J. Physiol. Endocrinol. Metab..

[38] Hancock CR (2008). High-fat diets cause insulin resistance despite an increase in muscle mitochondria. Proc. Natl. Acad. Sci. U.S.A..

[39] Lark DS, Fisher-Wellman KH, Neufer PD (2012). High-fat load: Mechanism(s) of insulin resistance in skeletal muscle. Int. J. Obes. Suppl..

[40] Eshima H (2017). Long-term, but not short-term high-fat diet induces fiber composition changes and impaired contractile force in mouse fast-twitch skeletal muscle. Physiol. Rep..

[41] De Wilde J (2010). An 8-Week high-fat diet induces obesity and insulin resistance with small changes in the muscle transcriptome of C57BL/6J mice. J. Nutrigenet. Nutrigenomics.

[42] Lees EK (2014). Methionine restriction restores a younger metabolic phenotype in adult mice with alterations in fibroblast growth factor 21. Aging Cell.

[43] Luo T (2019). Dietary methionine restriction improves glucose metabolism in the skeletal muscle of obese mice. Food Funct..

[44] Degens H, Koşar ŞN, Hopman MTE, De Haan A (2008). The time course of denervation-induced changes is similar in soleus muscles of adult and old rats. Appl. Physiol. Nutr. Metab..

[45] Paudyal A, Slevin M, Maas H, Degens H (2018). Time course of denervation-induced changes in gastrocnemius muscles of adult and old rats. Exp. Gerontol..

[46] Ballak SB (2016). Blunted angiogenesis and hypertrophy are associated with increased fatigue resistance and unchanged aerobic capacity in old overloaded mouse muscle. Age (Omaha)..

[47] Degens H, Alway SE (2003). Skeletal muscle function and hypertrophy are diminished in old age. Muscle Nerve.

[48] Plyley MJ, Olmstead BJ, Noble EG (1998). Time course of changes in capillarization in hypertrophied rat plantaris muscle. J. Appl. Physiol..

[49] Longchamp A (2018). Amino acid restriction triggers angiogenesis via GCN2/ATF4 regulation of VEGF and H2S production. Cell.

[50] Das A (2018). Impairment of an endothelial NAD+-H2S signaling network is a reversible cause of vascular aging. Cell.

[51] Minderis P, Fokin A, Dirmontas M, Ratkevicius A (2020). Hypocaloric low-carbohydrate and low-fat diets with fixed protein lead to similar health outcomes in obese mice. Obesity.

[52] Sjögren K (2001). Body fat content can be predicted in vivo in mice using a modified dual-energy X-ray absorptiometry technique. J. Nutr..

[53] Gargiulo S (2014). Evaluation of growth patterns and body composition in c57bl/6j mice using dual energy x-ray absorptiometry. Biomed. Res. Int..

[54] Kvedaras M, Minderis P, Krusnauskas R, Ratkevicius A (2020). Effects of ten-week 30% caloric restriction on metabolic health and skeletal muscles of adult and old C57BL/6J mice. Mech. Ageing Dev..

[55] Den Hoed M, Hesselink MKC, Van Kranenburg GPJ, Westerterp KR (2008). Habitual physical activity in daily life correlates positively with markers for mitochondrial capacity. J. Appl. Physiol..

